# iSelf-Help: a co-designed, culturally appropriate, online pain management programme in Aotearoa

**DOI:** 10.1186/s40900-022-00339-9

**Published:** 2022-02-21

**Authors:** Meredith A. Perry, Hemakumar Devan, Cheryl Davies, Dagmar Hempel, Tristram Ingham, Bernadette Jones, Susan Reid, Barbara Saipe, Patient Advisory Group members, Hazel Godfrey, Natalie Snaddon, Lily Morris, Nadine Puha, Bronwyn Haines, Alanna Irving, Matthew Brenycz, Technology Design Team, Paulien Fa’atafa, Kaylee Maclean, Siobhan Bulfin, Leigh Hale

**Affiliations:** 1 Centre for Health, Activity, and Rehabilitation Research (CHARR), School of Physiotherapy, University of Otago, Wellington, Aotearoa, New Zealand; 2 Tu Kotahi Māori Asthma and Research Trust, Wellington, New Zealand; 3 Wellington Pain Management Service, Capital & Coast District Health Board, Wellington, New Zealand; 4 Department of Medicine, University of Otago, Wellington, New Zealand; 5 Health Literacy New Zealand, Auckland, New Zealand; 6 Centre for Health, Activity, and Rehabilitation Research (CHARR), School of Physiotherapy, University of Otago, Dunedin, New Zealand

**Keywords:** Chronic pain, Co-design, Indigenous population, Participatory action, Patient and public involvement

## Abstract

**Introduction:**

Current best practice recommends group-based pain management programmes for long-term improvements in persistent pain-related disability. However, there are barriers for people to access in-person delivered pain management programmes in Aotearoa.

**Aims:**

To develop a co-designed, culturally responsive, online group-based pain management programme (iSelf-help) for people with persistent pain.

**Methods:**

A modified participatory action research (PAR) framework was used to co-design contents and cultural-appropriateness of iSelf-help. The PAR team included: (1) seven end-users living with persistent pain, who had previously attended an in-person delivered group pain management programme, (2) two pain management clinicians, (3) two health researchers, (4) two digital health experts, and (5) a health literacy expert. Five meetings were held with the PAR group and a Nominal Group Technique was used to rank order the preferred features of content delivery. In parallel, to ensure cultural appropriateness of iSelf-help, three focus groups (n = 15) were held with Māori (the Indigenous population of Aotearoa) living with persistent pain in collaboration with a Māori community health trust. All contents were reviewed by a Māori Health literacy expert and core contents were translated into Te Reo (Māori language). All contents were finalised by iterative discussion among the PAR team and consultation with Māori stakeholders. The preliminary version of iSelf-help was pilot tested with the PAR group participants and Māori community members living with persistent pain and their feedback was included. The iterative co-design process occurred over a period of nine months.

**Results:**

The finalised version of iSelf-help included a total of 130 resources organised in to 12 content relevant online modules plus a dedicated welcoming page and an online community forum. Each module included: short videos, animations explaining main concepts, patient stories, written content to accompany visual content, podcasts of relaxation techniques, illustrated texts, and evidence-summaries. A dedicated module of videos demonstrating cardiovascular and strengthening exercises of varying intensity was also included.

**Conclusions:**

This is the first co-created, culturally appropriate, on-line group pain management programme for people with persistent pain, developed in Aotearoa. The next step is to evaluate the clinical and cost-effectiveness of iSelf-help compared to in-person delivered pain management programme.

**Supplementary Information:**

The online version contains supplementary material available at 10.1186/s40900-022-00339-9.

## Plain English summary

Pain management programmes delivered in a group format are best practice to support people living with persistent non-cancer pain to live well. Some people can find accessing these programmes hard due to lack of referral, transportation costs and lack of trained health professionals. Further, people from Indigenous and non-Western backgrounds are poorly represented in these programmes despite having a high prevalence of persistent pain. One way of improving access is delivering services via technology. We aimed to co-design an online version of an existing hospital-based pain management programme (iSelf-help) and to ensure cultural appropriateness of the iSelf-help for Māori. Māori are the Indigenous population of Aotearoa (an accepted Māori word to describe New Zealand). We used a modified participatory action research (PAR) framework for our co-design process. This framework actively encouraged people with lived experience of pain and community partners to have a voice in the content design. The PAR team included: (1) seven end-users living with persistent pain, who had previously attended hospital-based pain management programme, (2) two pain management clinicians, (3) two health researchers, (4) two digital health experts, and (5) a health literacy expert. Five meetings were held with the PAR team. We used a Nominal Group Technique, whereby PAR team members ranked their preferences on content design and delivery, until concensus was reached. In parallel, three focus groups (n = 15) were held with Māori living with persistent pain in collaboration with a Māori community health trust. All contents were reviewed by a Māori Health literacy expert and core contents were translated into Te Reo (Māori language). The finalised version of iSelf-help included 130 resources, tested for accessibility, organised in to 12 online modules plus a dedicated welcoming page and an online community forum. Each module included: short videos, animations, patient stories, podcasts of relaxation techniques, illustrated texts, and evidence-summaries. This is the first co-created, culturally appropriate, on-line group pain management programme for people with persistent pain, developed in Aotearoa. We are currently evaluating if iSelf-help is acceptable to users, and clinically and cost effective as compared to the hospital-based pain management programme.

**Supplementary Information:**

The online version contains supplementary material available at 10.1186/s40900-022-00339-9.

## Background

Persistent non-cancer pain is a significant health problem affecting individuals and society. In Aotearoa (an accepted Māori word to describe New Zealand—meaning land of the long white cloud), the annual prevalence of persistent non-cancer pain in the general population is 19.6% (2019) [1]. However, persistent non-cancer pain disproportionately affects Māori (the Indigenous population of Aotearoa) who have a pain prevalence of 23.2%[1]. The health costs from persistent non-cancer pain conditions is estimated to reach 24 billion NZD by 2048 [2]. A number of systemic challenges [3] contribute to the rising economic and societal costs of persistent pain conditions. These challenges include delays to initial diagnosis, long waiting times for referral to secondary and tertiary pain services, lack of understanding and validation from healthcare professionals [3], limited resources for multidisciplinary tertiary pain services [3], and barriers for Māori [4] and other minority population groups (e.g. Pacific and Asian peoples) to receiving referrals for tertiary pain services [5]. If referred and accepted into a pain service, lack of acknowledgement of spiritual beliefs and poor clinician-patient communication were cited as barriers to optimal pain management for Māori and Pacific patients [6].

Māori adults are at 1.4 times higher risk of reporting persistent pain than non-Māori adults [1]. Māori also experience inequitable health outcomes in other long-term health conditions such as diabetes, cardiovascular diseases, and cancer [7]. While factors across the social determinants of health create these health inequities, systemic racism in health care services, lack of culturally responsive care acknowledging Māori health beliefs, and barriers to access of primary care services perpetuate these inequities [8]. Māori living with persistent pain report previous experience of racism in health care services [9], an overreliance on prescription of pain medications as the primary pain management strategy [4], lack of culturally responsive care, and limited specialist referrals from primary care services [4] as barriers contributing to under-representation of Māori in tertiary pain services [5, 6].

Te Tiriti of Waitangi (Te Tiriti) or Treaty of Waitangi is the founding document of New Zealand (NZ) and guides the NZ Ministry of Health’s and those who work within the health sectors, obligations to Māori, including to address inequities and ensuring optimal health outcomes for Māori [10]. The four main goals underlying the Ministry of Health’s approach to Te Tiriti are Mana whakahaere (Māori stewardship), Mana motuhake (Māori self-determination), Mana tangata (achieving Māori equity), and Mana Māori (enabling Māori customary rituals) [10]. These goals are achieved by working in partnerships with the health and disability services to ensure the health services are providing culturally appropriate care to achieve equity for Māori [10].

In line with the obligations of Te Tiriti, one way of addressing inequities in pain management is to partner with end-users and communities to co-design culturally responsive service delivery and resources. Evidence for the effectiveness of co-design approaches in health services research suggest improved uptake and community ownership [11]. Despite the potential benefits of meaningful engagement with end-users and communities, there is clear consensus on lack of reporting of *‘how’* patient engagement occurred in previous clinical trials [12]. Our ongoing non-inferiority clinical trial (iSelf-help trial) evaluates the clinical and cost-effectiveness of a co-designed, culturally appropriate online pain management programme (iSelf-help) compared to in-person delivered programme. The main aim of this paper is to describe *‘how’* the co-design processes were conducted in developing iSelf-help. By clear reporting of methods used for our engagement, it may assist similar intervention developers who are considering using a participatory approach.

## Methods

### Study design

**Fig. 1:**
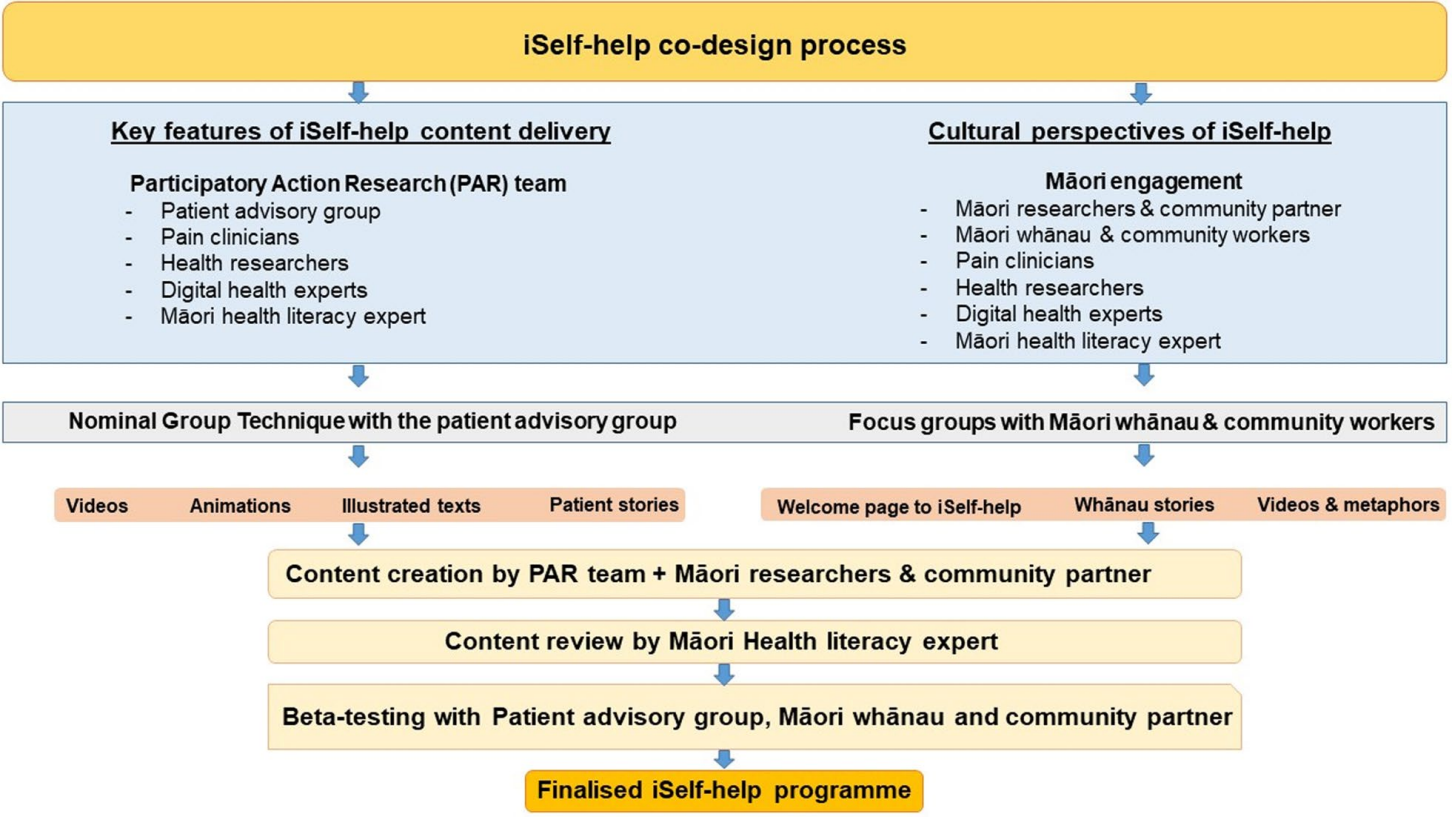
An overview of the parallel co-design processes of iSelf-help programme development guided by a participatory action research framework

We used a 5-step modified participatory action research (PAR) framework to develop, evaluate and implement iSelf-help; this is a recommended approach for developing rehabilitation interventions [13]. Participatory action research (PAR) is a collaborative, reflective, and transformative research paradigm in which patients and other end-users are actively involved from the inception to implementation phases [14]. The teams experiences of co-designing iSelf-help were reported in a separate paper [15]. The five steps of PAR are (1) Agenda setting, (2) Design of iSelf-help, (3) Implement/Evaluate (4) Dissemination and (5) Sustainability. This paper describes the processes (Steps 1 & 2 of PAR) and who was involved in co-designing the iSelf-help online intervention. Of particular relevance to the co-design elements was the inclusion of past pain management group patients as part of our patient advisory group (PAG) and local Māori community led by our Māori community partner (CD) from Tu Kotahi Māori Asthma and Research Trust and two senior Māori researchers (TI and BJ). The former group was essential for the transition from in-person to online format. The latter group was responsible for cultural appropriateness. In consultation with our Māori researchers (CD, TI and BJ), it was decided to hold these meetings separately as most Māori patients have not been referred to a pain management programme. An outline of these parallel co-design processes is illustrated in Fig. 1.

The Guidance for Reporting Involvement of Patients and the Public—GRIPP2 checklist was used to report the findings of this co-design study (See Additional file 1) [16].

### Agenda setting

The PAR team initially met and decided upon the key objectives and research priorities in developing, evaluating and implementing the iSelf-help intervention. The initial meeting also developed group communication and respect guidelines, assigned individual responsibilities within the team, and setting up of a common communication channel for ongoing dialogue and problem solving. The PAR team included:

1. A patient advisory group (PAG) consisted of seven participants with persistent pain, 20–60 years of age, (6 women, 1 man, 1 Māori), who had recently completed an in-person PMP at at one of the three tertiary pain managements service in Aotearoa. The in-person PMP is a group-based programme comprising of a weekly 4-h session over 12 weeks. The programme is offered three times every year facilitated by an inter-disciplinary team of physiotherapists, psychologists, an occupational therapist and a pain medicine specialist.
  *Recruitment of PAG members*: We intended to recruit 10 participants. Two pain management clinicians (DH and BS) identified potential participants from previous PMP groups over the last four years. These potential participants were recent completers of the in-person PMP. The clinicians initially contacted these ex-patients and sought their interest to be involved in the research. If interested, their contact details were forwarded to the research team. The research team members contacted the ex PMP patients and invited them to join the PAR team for the co-design of the online PMP. Out of 11 who were invited, eight accepted the invitation (20–60 years of age, 7 woman, and 1 man, 1 Māori). One participant who attended the first meeting was unable to attend subsequent meetings due to personal reasons. Reasons for declining to participate included the time commitment (n = 2) and expense of travelling to the University (n = 1).
2. Two physiotherapists (DH and BS) from the CCDHB pain management service. We have previously engaged with these clinicians while conducting a qualitative study evaluating the impact of introducing self-management concepts in to their in-person, pain management programme [17].
3. Two academic health researchers (HD and MP), who are core research team members.
4. Two Digital health experts (KM and PF) from Melon Health™. Melon Health™ is a Aotearoa based digital company experienced in providing innovative technological management support for people living with long-term health conditions [18, 19].
5. A Māori health literacy expert (SR). The health literacy expert joined a few months after the co-design process as the need for health literacy review of online contents was recommended following Māori community engagement.

### Design of iSelf-help

As outlined in Fig. 1, the co-design processes occurred simultaneously with the PAG members of the PAR team and Māori living with persistent pain (via Māori community meetings called hui) to ensure cultural appropriateness of iSelf-help.

#### Expectation setting

Based on the discussions with the PAG members, to create a dose comparable intervention to in-person PMP, it was agreed that the core educational messages of the 12-week in-person PMP and structural elements (exercises and moderated group discussions) were to be retained. This decision was also discussed and agreed with members of the Māori community hui. As PAR team members, representatives from Melon Health™ attended the first, and most subsequent PAR group meetings and Māori community hui to ascertain content delivery ideas as well as moderate expectations. Content delivery limitations included elements such as interactive video games due to funding restrictions and any ideas which required significant alterations to the Melon Health™ online platform template.

#### Nominal group technique

A Nominal Group Technique [20] was used to seek ideas on how to generate contents and derive group consensus with the PAR team. This technique enables collective problem solving [21] while ensuring equal contribution from each group member, with minimal influence of researchers [21]. Five in-person meetings were conducted including the agenda-setting meeting with the PAR team from October 2018 to May 2019. Each meeting had 5–6 PAG members, clinicians, digital health team members and was facilitated by two researchers (HD and MP).

We followed these five stages of Nominal Group Technique to guide the PAR group meetings for content generation:

1. *Introduction and explanation of topics of discussion*: Each session started with an introduction of each person and their role in the project, and topics for discussion.
2. *Silent idea generation (individual)*: Each session had three discussion topics based on the in-person PMP sessions. Participants were given an individual idea generation sheet to write their ideas for the *two key questions* relevant to those three discussion topics.
3. Content-related question
  - What was the most valuable aspect of learning/content from these sessions? What suggestions do you have for additional information or perspectives (i.e., cultural/spiritual or breadth/depth of content)?
4. Delivery style related question
  - How do you envision this content being delivered in an engaging/interactive way? What do you think the most beneficial formats/features for making sense of and using this information in an online medium would be (For example, consideration of learning styles)?
5. *Individual member discussion of their ideas*: Each participant from the PAG shared their ideas, which were documented on a whiteboard by a researcher.
6. *Group discussion of collated ideas*: Once all individual ideas were captured in the whiteboard, participants as a group shared their views on the collated ideas. Based on the discussion, some ideas were merged and re-organised. Providing the opportunity for every individual to speak and contribute to the discussion was considered important.
7. *Re-ranking of collated ideas*: Participants were provided an individual ranking sheet to document their top priorities from the collated ideas. We frequently ran out of time at the end of each meeting, therefore we asked participants to re-rank ideas prior to the next meeting. They were also encouraged to send additional responses via e-mail.

All the PAR team meetings were held in a University meeting room and were audio-recorded and key points were noted. The summary results were presented back to the group in the following meetings for further feedback and consensus. The meetings were scheduled for 2.5 h with refreshments. The refreshments provided an opportunity for the participants to informally chat and share their ideas with each other. They also served as a sign of hospitality and respect. A grocery or petrol voucher was provided at each meeting to PAR team members for their time and parking costs.

#### Content delivery

**Fig. 2:**
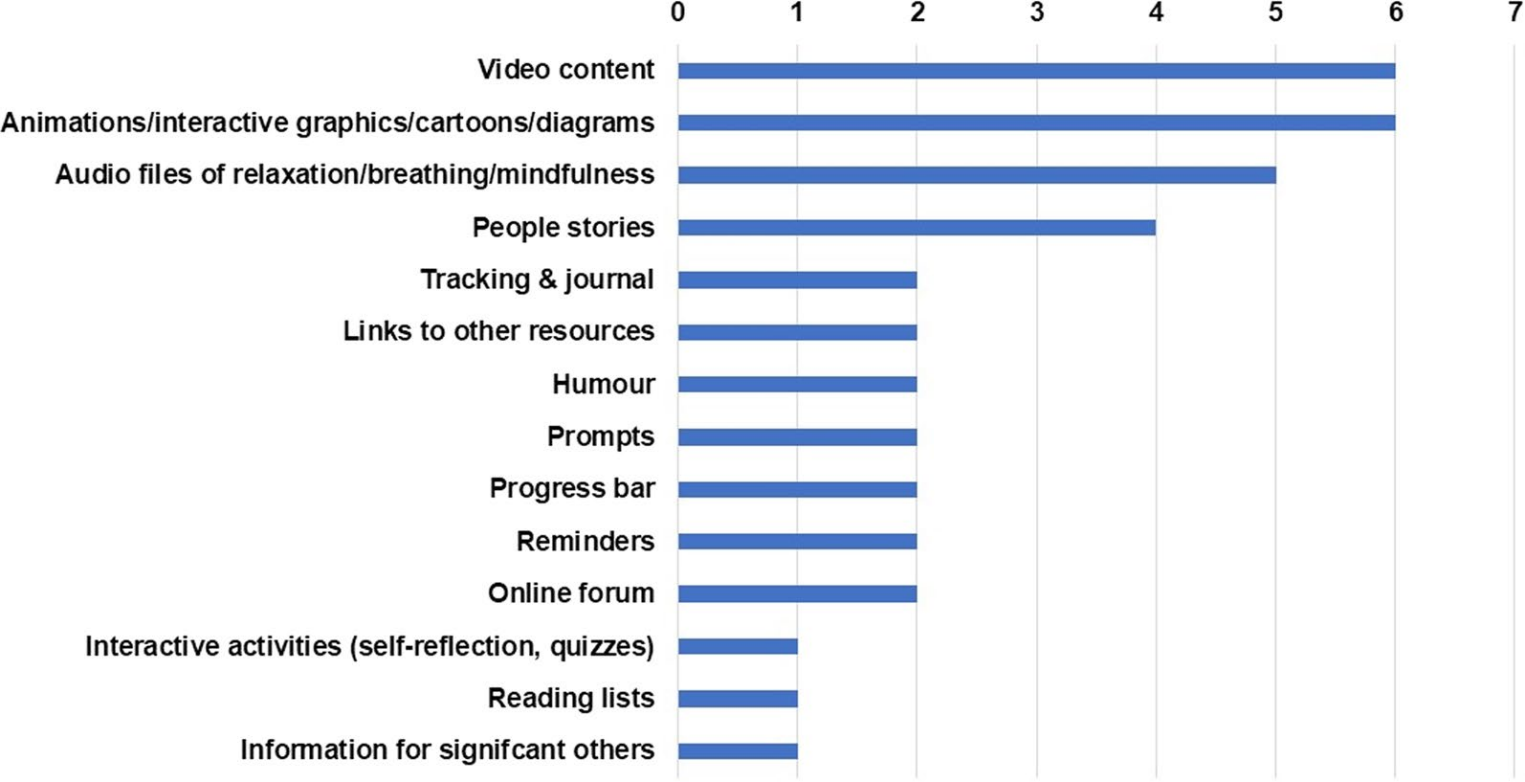
Summary features requested by patient advisory group members

The rank order of content features that PAG members requested were shown in Fig. 2.

*Video content*: Participants wanted online information predominantly delivered via videos or visuals (Fig. 2). They suggested having a brief *introductory video* at the beginning of each online module by a clinician explaining an overview and key objectives of each module and a *main video* summarising the key educational information of that module. Participants also requested the need for exercise videos with a progressing level of intensity over the 12 online modules.

**Fig. 3:**
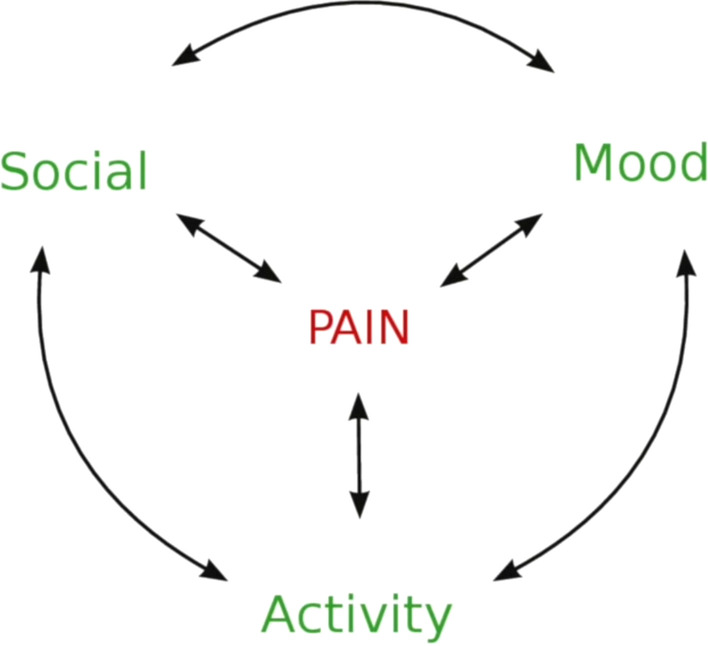
Patient Advisory Group member wanted interactive texts explaining the inter-connected impact of pain on mood, activity and social participation. This figure is an example of how pain was perceived to affect and be effected by social activities, activities of daily living, and mood

*Interactive texts*: Participants specifically expressed the need for visual representation of key concepts of each module (Fig. 2). They wanted visuals explaining the inter-connected elements and suggested using one overarching visual (woven) throughout the module. Some specific examples included explaining the inter-connected effect of pain on activity, mood and social connection as illustrated by a participant below in Fig. 3.

**Fig. 4:**
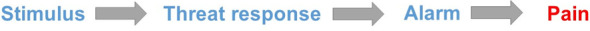
Patient advisory member feedback to co-create an animation explaining sensory nervous system, threat response and pain experience as an output. They discussed how a stimulus leads to the threat response, which causes alarm and consequently the perception of pain

*Animations*: The use of animations for explaining key pain biological concepts such as difference between acute versus persistent pain, the sensory nervous system pathways and the role of neural synapses and pain memory were requested. This material was considered helpful for both the person living with pain and their whānau (family member and significant others), as a way of explaining what they are going through. A participant suggested the need for an animation explaining the mechanism of how stress response leads to pain (Fig. 4).

*Metaphors*: PAG members recollected some of the useful metaphors that they learnt from the in-person PMP as a useful way of understanding and retaining information. Particularly, participants shared making sense of threat response using an analogy of “Tiger in the room” [22]. Resources from the Explain Pain book [23], including Professor Moseley’s story of a snakebite (understanding sensory processing of pain) and the Baker’s story (as an example of pain memories triggering a threat response, which was represented by smell of the bread triggering a pain response) were recommended as valuable learning resources to be included.

*Relaxation podcasts*: Participants discussed the benefits of mindfulness relaxation exercises during the in-person PMP. To make these more accessible, participants suggested having audio recordings of relaxation exercises and sound clips downloadable in an MP3 format.

*People’s stories*: Participants noted that sharing and listening to ‘similar others’ during their in-person PMP had been hugely beneficial for reinforcing their current helpful self-management strategies and for learning new ways of managing their condition (Fig. 2). Thus, they requested that people’s pain journeys be an aspect of the online modules. Importantly, they wanted the people in these stories to be ‘*relatable*’ to reflect the different backgrounds, ages and cultures of people who experience persistent pain.

*Peer-support *via* peer-support facilitator and online community forum*: Participants expressed the need to mimic the shared understanding and learning (‘Mastery experiences’) that they derived from the in-person PMP using an online community forum. They endorsed the researchers’ suggestion of having a peer-support facilitator with lived experience of pain to facilitate the discussions in an online community forum. A weekly interactive videoconferencing discussion with a peer-support facilitator was perceived as necessary to create a safe space for learning and sharing from each other.

*Structuring of contents in an online module*: To keep the online modules more interactive and user-friendly (i.e. to maximise engagement), participants suggested having a progress bar, and providing sign posting in terms of key contents of each module. To check understanding and reinforce learning, they also suggested having short quizzes at the end of each module.

### Cultural perspectives of iSelf-help

In keeping with the principles of Te Tiriti, we aimed to incorporate Māori cultural perspectives within iSelf-help and conducted three Māori community-based focus groups (n = 10, participants were 30–70 years of age, 2 male) in parallel to the PAR group meetings. While one of the PAG members identified as Māori, they did not feel comfortable as an individual to be responsible for representing a Māori worldview. In addition, the decision to hold these meetings separately was guided by our senior Māori researchers to ensure the participating whānau were feeling safe to share their views and because the focus of these meetings were different. Two of the focus groups were held with Māori adults living with persistent pain and their whānau and one with kaiāwhina (Māori community support workers) supporting Māori and their whānau (Fig. 1).

*Cultural considerations and procedures*: Our engagement was underpinned by Māori centred research principles [24]. The key principles guiding Māori centred research are recognising Māori values and customs, following tikanga (Māori customary) processes throughout the research project, meeting the aspirations of the participants and meaningful engagement with the community [25]. In keeping with the Māori centred research principles, the senior Māori researchers of our team (TI and BJ) with expertise in Kaupapa Māori (Māori way of doing) research [25] and our Māori community partner (CD) guided the hui process. All hui were held kanohi ki te kanohi (in-person) with local iwi whenua (Indigenous people) and Kaumātua (respected Māori elders).

*First hui (focus group)*: The first hui was primarily aimed at establishing meaningful relationships (Whakawhanaungatanga). Initially, both researchers and participants introduced themselves through mihi whakatau (formal greeting and introductions). We asked about participants’ experiences of persistent pain and their self-management strategies. As none of the Māori participants had previously attended an in-person PMP, the hui discussions were informed by showing them a culturally tailored online resource for Māori (https://depression.org.nz/maori/). Our community partner (CD) led the hui, supported by a researcher (HD). The meetings were held in a community centre (Tu Kotahi Māori Asthma and Research Trust). All participants were provided kai (refreshments) and a grocery voucher as an acknowledgement for their contribution. The meeting was audio-recorded and a written summary of meeting outcomes was noted.

*Second hui (focus group)*: To start, a summary of the first hui was presented and any further feedback was sought. The same participants were shown some of the key features of the online programme. This included snapshots of the online programme, community forum, some of the resources in the online modules and a draft overview of the welcoming page website to the programme. In small groups, participants shared their views and ideas about the online programme. Field notes and Post-it® notes were used to collect feedback. The Māori (TI and BJ) researchers and community partner (CD) led the hui and non-Māori researchers (HD, LH and MP) assisted with the data collection process by taking field notes during small group discussions. All sessions were audio-recorded to capture the main discussion points.

*Recommendations*: From both hui, there was a consensus to focus on not only the individual with persistent pain but also their ‘whānau’ (family and significant others). The feedback from the Māori section of the website (https://depression.org.nz/maori/) was overwhelmingly positive and they recommended having a similar welcoming page including a short video of a Māori personality welcoming people to the pain programme along with karakia (prayer) and whakatauākī (Māori proverb). The use of whānau stories of pain was suggested as a way of helping others to learn and care for each other. The use of simple language with minimal jargon using both Te Reo (Māori language) and English was recommended.

## Results

### Content generation

Based on the feedback from PAG members and Māori engagement, most of the existing contents in the 12-week in-person PMP were retained and new online contents were created as short videos, animations and illustrated texts in collaboration with the digital health team. The two pain management clinicians of the PAR team (DH and BS) coordinated the content generation process along with input from the researchers. The researchers (HD and MP) created evidence-based summaries of relevant topics in collaboration with clinicians. The Māori researchers and community partner led the cultural appropriateness aspect with input from researchers and clinicians. Some of the existing online pain management resources (e.g. websites, apps and YouTube videos) were also included in iSelf-help, which were informed by previous evaluations conducted by our team [26, 27] and from a systematic review [28]. Accessibility evaluation and checks, which includes red/green colour blindness testing, were conducted as part of the digital health team’s design process. We present below some of the key features of iSelf-help and the underpinning iterative processes.

### Welcoming page to the online programme

**Fig. 5:**
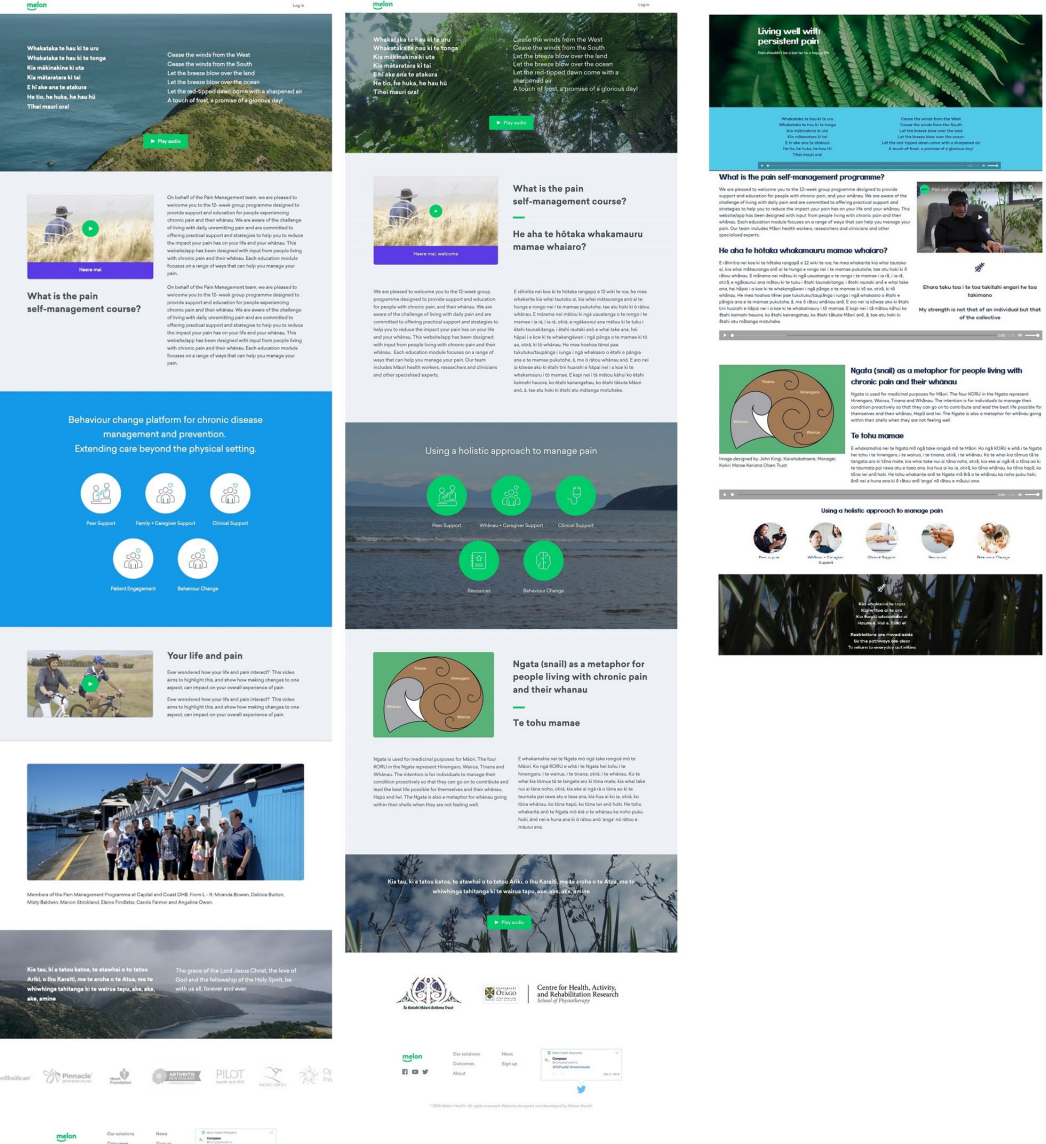
The figure shows three versions of the welcoming page **a** initial version with a cape resembling Cape Reinga, **b** second version with suggested pictures, and **c** final version with karakia, whakatauākī (Māori proverb), audio and English/Te Reo version

Based on Māori participant recommendations, a draft welcoming page for the online programme was created by the design team including natural imagery and English/Te Reo texts. There are three key features. First, as shown in Fig. 5, we included a picture of a cape or headland in the first draft of the welcoming page (Fig. 5a) which resembled “Cape Reinga” situated at the northern tip of North Island, Aotearoa. However, this cape is considered by Māori to be a point where dead souls enter into the underworld, and thus the Māori community partner and our health literacy expert, who is also Māori, did not think this appropriate as a welcoming picture. In the second iteration (Fig. 5b), the Māori community partner liaised with the Māori participants and together they collected photos of native bush imagery more appropriate for use in the welcoming page.

Second, opening and ending karakia (short prayers) were also included in the home page as suggested by the Māori community partner. There was, however, disagreement with one of the karakia suggested as it had reference to a specific religious ideology and consensus was that a different karakia which was still spiritual be used instead.

Third, similar to depression.org.nz, we attempted to have a Māori personality fronting the home page and welcoming the whānau to the online programme. However, this was not possible in our time frame, so we used clips from whānau stories as an introductory video to welcome people and whānau to the online programme instead (https://www.melonhealth.com/programs/pain/).

### Ngata as a metaphor for explaining holistic impact of pain on whānau

**Fig. 6:**
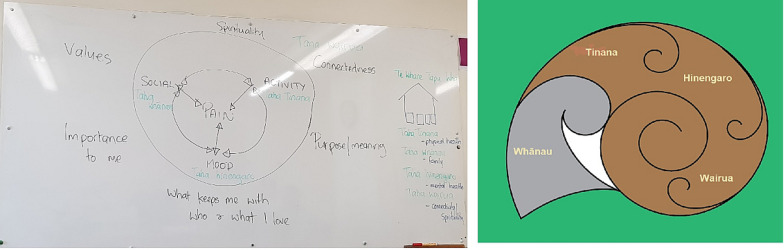
The figure **a** shows clinicians’ way of explaining the impact of pain and **b** the resultant output from iterative co-design process—“Ngata (snail)” as a metaphor for explaining the holistic effect of pain on Whānau (family and significant others), Tinana (physical wellbeing), Hinengaro (psychological wellbeing) and Wairua (Spiritual wellbeing)

The pain management clinicians and our Māori community partner initially met to co-produce a visual explaining the impact of persistent pain on mood, stress, physical activity and social participation (Fig. 6a). This is similar to the biopsychosocial model of pain management [29], however the biopsychosocial model does not acknowledge the importance of spirituality, an important aspect of pain management for Māori [9]. Based on the suggestions that metaphors and images provide deeper insights of Māori world view [30], our Māori community partner in collaboration with a Māori designer, created the “Ngata” (snail) image as a metaphor for explaining our model that represents the holistic impact of pain (Fig. 6b).

Ngata refers to a snail in Te Reo. It is used as a Rongoā (traditional medicine) in Māori culture to cure respiratory ailments. The metaphor Ngata was co-produced over several iterative discussions with our Māori community partner and clinicians to help explain the impact of pain on whānau (family and significant others) based on the “Te Whare Tapa Whā” model of Māori health [31].

In Te Whare Tapa Whā, Māori views on health and wellbeing are holistic interconnecting the four essential tenets of life of Tinana (Physical health), Hinengaro (Psychological health), Wairua (Spiritual health), and Whānau (Family and significant others wellbeing) [31]. Our Māori community partner came up with the explanation that whānau living with persistent pain could go within their shells when experiencing pain (Fig. 6b) and the aim of the programme was to assist whānau to come out of their shell “to live life” by providing tools and strategies to foster living well with persistent pain.

### A brief outline of online modules

Each online module had an *introductory video* (30 s in duration) by a pain management clinician leading the module and an *explanatory main video* (4 to 5 min in duration). The supporting resources included one or two *interactive texts* illustrating the educational concepts and metaphors. Each module also had a *relaxation audio podcast*, available in a downloadable MP3 format. As participants wanted to read the transcripts of videos or relaxation podcasts, the links to transcripts were also presented. We included *evidence-based short summaries* of interventions (e.g. Mindfulness) and fundamental concepts (e.g. Fatigue) in applicable/relevant modules, as recommended by PAG members.

**Fig. 7:**
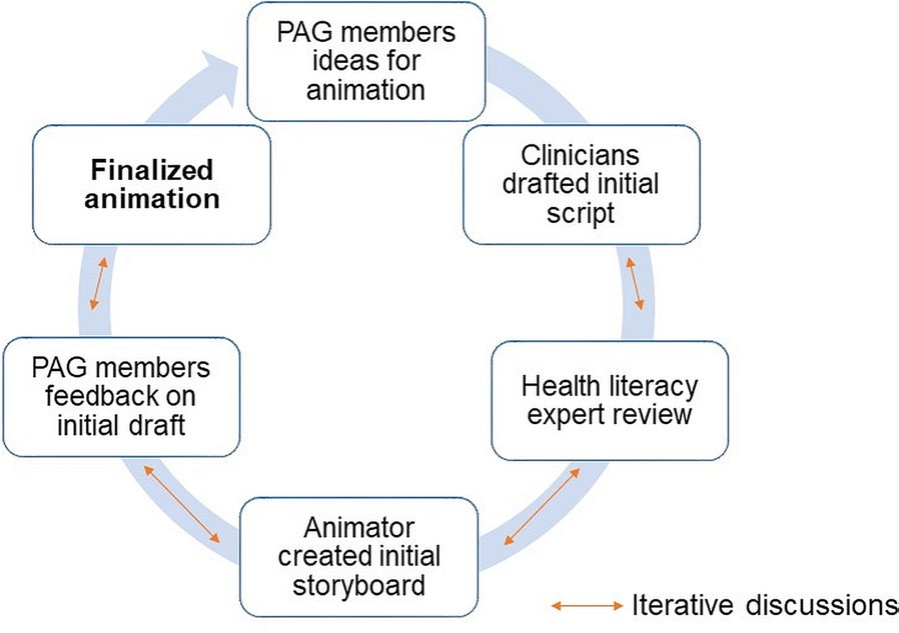
Iterative co-design process in creating animations for iSelf-help

*Animations*: In addition to the aforementioned resources, three animations were co-designed by PAG members, pain clinicians, an animator and the health literacy expert. The iterative process is explained in Fig. 7, initially, PAG members reviewed several types of model animations and informed the look and feel of animation and concepts that could be animated. Then, the clinician created a draft animation script. The health literacy expert subsequently reviewed the initial version of the animation script and the animator created a draft storyboard. After iterative discussions amongst the clinician, animator and health literacy expert, an initial version of the animation was created. The PAG members commented and further revised the animation before it was finalised (Fig. 7).

**Fig. 8:**
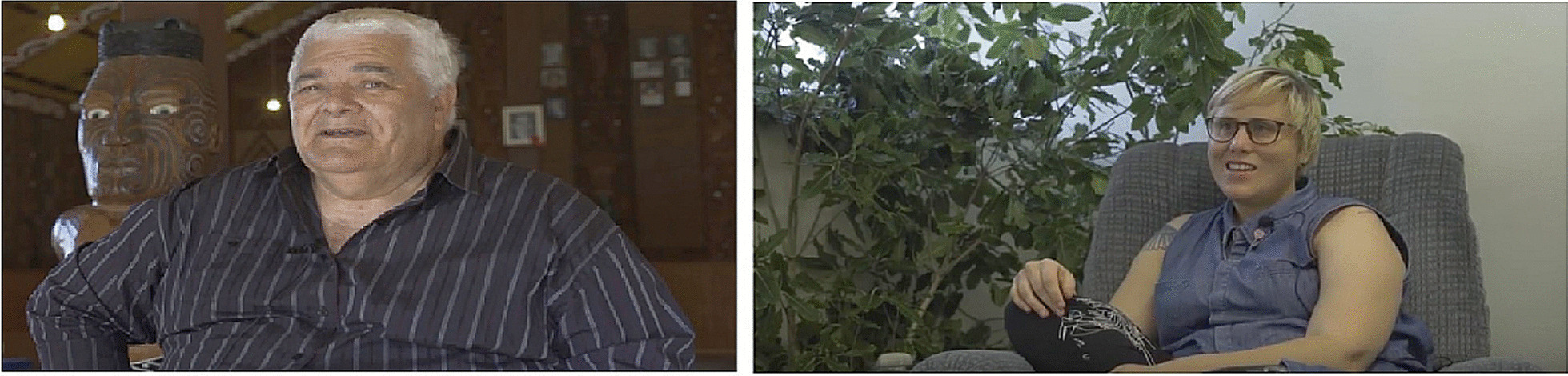
**a** Filming of Māori participants was held in a local Marae and **b** the filming of PAG members was held in a hospital room

*Patient stories*: Both PAG and Māori community members were invited to share their experiences of pain management in general and some of the key messages from completing the in-person PMP. Twelve participants were filmed (eight from PAG members and four from Māori whānau). The whānau filming took place in a Marae (Māori meeting house) (Fig. 8a) and the PAG filming was done at the hospital (Fig. 8b). To put participants at ease while filming, the hospital room was arranged with native plants and a comfortable chair as shown in Fig. 8b. A dedicated interview guide was sent to participants prior to the video interviews (See Additional files 2 & 3). The video clips of interviews were time-stamped to match key information and stories to the 12 modules independently by clinicians (DH and BS) and researchers (HD and MP). Once the placement of these elements was agreed upon they were embedded into relevant online modules.

**Fig. 9:**
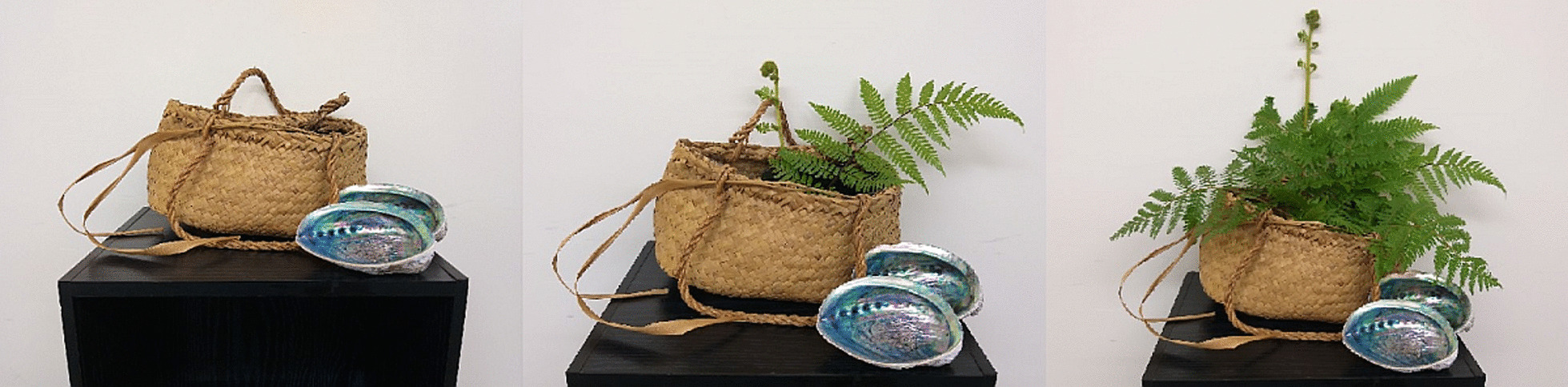
A kete (basket of knowledge) was used as a visual metaphor for gaining tools and confidence in managing pain with completion of each iSelf-help online module over 12 weeks

*Kete (basket of knowledge)*: Kete refers to a basket in Te Reo. As a way of metaphorically illustrating the progress of each online module, a kete was pictured to highlight that the learnings and knowledge gained from each module (Fig. 9). The ferns symbolise, and signal to the person in pain, increasing tools and confidence for managing their persistent pain by way of ferns blossoming in the kete and thus filling the basket with knowledge as the programme progressed.

### Health literacy review and Te Reo translation

Based on the feedback from Māori participants, the Māori health literacy expert (SR) reviewed all the content including scripts for animation, videos and interactive text in the online modules and subsequent revisions were incorporated in collaboration with the PAR team clinicians. The resources were not developed for any specific literacy levels. Instead SR looked at all aspects of the text: purposes, language, genre format, engagement with the audience, grammatical constructs such as active or passive voice, and framing of the material in the context of the whole project. Prior to Beta-testing of the online programme, SR was granted access to all the online resources so was able to see the context in which the texts were being used and how users had choices in relation to how they navigated the programme. From there SR made changes to the text to ensure consistency across all written texts in the programme. Due to lack of funding, only the core resources, as identified by PAR team clinicians, were translated to Te Reo by a professional translation service.

### Beta testing

A final round of testing of the online programme, the Beta testing, was undertaken by both the PAG members and the Māori community members. They were provided access to the prototype version and encouraged to go through the content of the online programme using both the website and their smartphone app versions. After two weeks, a follow-up feedback session was held for both PAG and Māori community hui members. For those members who were unable to take part in the in-person session, written feedback was encouraged and included in the analysis.

Participant feedback were summarised (see Supplementary material) and most suggestions were included in the final version of the online programme. We were unable to include some suggestions (e.g. progress bar for each module, short quizzes and a FAQ section) due to limited time and budget constraints, as well as technical flexibility constraints of the online platform. Some common themes from Beta testing were: (1) The need for an instructional video of *‘How to use the app/online platform’* so that the users can navigate various features of the online programme (iSelf-help) and the types of supporting resources, (2) To arrange the order of resources in each app to follow a consistent order, in which the introduction and main explanatory video were displayed upfront followed by other supporting resources, and (3) To internally link a number of relevant resources within and across online modules.

The Māori community members requested the need for an audio playback for the karakia (prayers) as they felt it would enhance accessibility of the written information in the welcoming page to the programme. These members also requested more Māori visuals on the welcoming page. All participants commended the use of Te Reo in the online modules.

## Final version of the online programme—iSelf-help

**Fig. 10:**
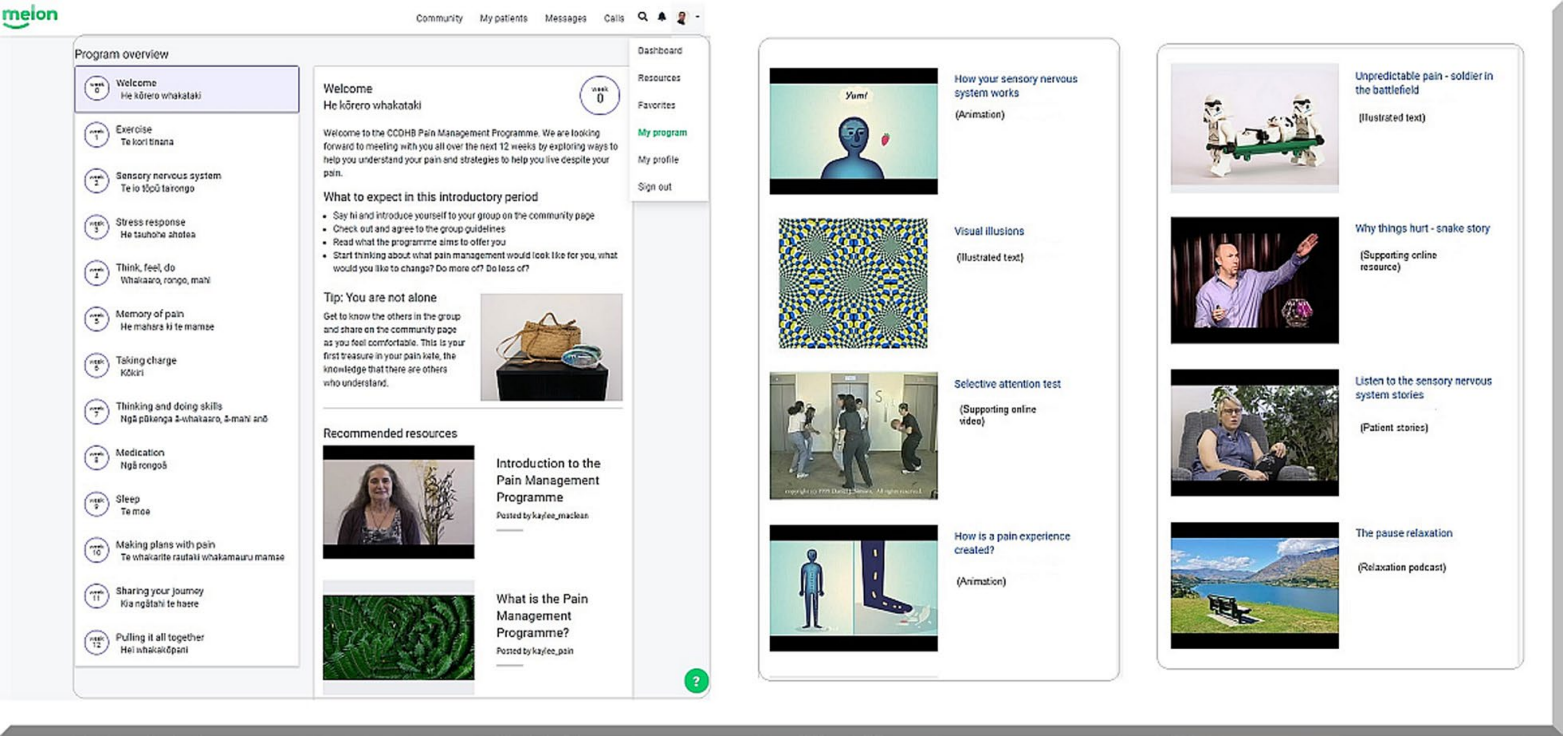
**a** The 12 online modules of iSelf-help with each module objectives, tips for the week and recommended resources including introductory videos, **b** animations, illustrated text information, supporting online videos and **c** patient stories and relaxation podcasts

The finalised version of iSelf-help included 130 resources in total organised in 12 online modules (Fig. 10). A dedicated welcoming page was created for the study (https://www.melonhealth.com/programs/pain/). A dedicated exercise module including videos demonstrating cardiovascular and strengthening exercises of varying intensity was also included. The other 11 modules included: short videos explaining the concepts, animations, patient stories, written blurbs, illustrated texts and evidence-summaries (Fig. 10). Each module is opened sequentially over 12 weeks. At the beginning of each week, participants go through the resources within each module guided by a paid peer-support facilitator. The peer-support facilitator is one of our PAG members, whom expressed interest in this role. Later in the same week, participants have a 60–90 min videoconferencing session with clinicians to go through the educational contents of the module and to ask further questions related to the module. A dedicated community forum as part of the programme was created to facilitate peer-to-peer interactions and sharing of information during the 12-week programme. The community forum was monitored by a health coach (from the digital health company) to ensure the safety of messages posted in the forum.

## Discussion

There is a paucity of literature on co-design methods for developing group-based pain management programmes. The primary purpose of this paper was to describe the processes of *‘how’* and ‘*wha*t’ we co-designed for a clinician-supported, group-based, online PMP (iSelf-help) that is culturally appropriate for Māori living with persistent pain. We used a PAR framework to guide our co-design process and Māori engagement. A Nominal Group Technique and a collaborative, culturally appropriate focus group method informed our co-design engagement with the PAG members and the Māori community members living with persistent pain respectively. The co-design and development of iSelf-help contents was a complex project which took several different teams of people working collaboratively as PAR team members over nine months (September 2018 to May 2019). As reported previously [15], this necessitated ongoing commitment, negotiation of mismatched expectations, and a coalition to be successful as a PAR team [15]. We are currently evaluating if iSelf-help is acceptable to users, and whether the programme is clinically and cost effective as compared to the hospital-based pain management programme [32]. In the following discussion section, we highlight some of the key learnings from our iSelf-help co-design processes.

*Challenges of using Nominal Group Technique*: Although we used a Nominal Group Technique to brainstorm ideas and achieve group consensus among the PAG members, we found it was effective when most of the PAG members were present in-person during the meeting. Some PAG members were unable to travel to attend the meeting, so attended via Zoom. This made it challenging to efficiently facilitate group discussion and derive consensus between those present in-person and those online. Also, during the idea generation phase of the Nominal Group Technique, we asked advisory members to write their initial thoughts on paper and some participants found it challenging to accurately capture their thoughts on paper. We managed this challenge via assisting them during that writing process by encouraged them to verbalise their thoughts which we then recorded in writing. Another common challenge we encountered was the time constraints associated with running these group meetings. Most meetings went over time and it took us about 2–2.5 h for each meeting. Although we reimbursed the travel cost of the PAG members, the increased time commitment was not expected and our reimbursements could be considered inadequate [34]. However, having refreshments during the meeting helped break-up the meeting and encouraged informal conversations between participants and research facilitators.

*Peer-support in an online medium*: Both the PAG members and the Māori participants emphasised the value of peer learning and support in the form of short video stories capturing experiences of people living with persistent pain using various self-management strategies. Video stories were proven to be useful patient education tools and improve health-related behaviours in people with asthma [35] and diabetes [36]; however, the potential for video stories fostering health behavioural change in people with persistent pain remains less explored [37]. While we prepared a semi-structured interview guide for the PAG members and the Māori participants to share their stories, we recommend future studies to adopt creative methods such as storytelling [38] and art-based approaches [39] for providing deeper insights of personal stories of living with persistent pain, as well as ensuring a wide range of stories and experiences from a diverse group of people.

*Different types of content meant the extent of co-design varied*. In total 130 resources were loaded onto the iSelf-help programme. However, due to the number and volume of content co-designed increasing more than anticipated, the extent of co-design varied for individual content. For example, the welcoming page to the programme and the Ngata as a metaphor for the impact of pain on Māori whānau were co-created with whānau and a Māori community researcher. On the other hand, for the three animations, although the PAG members generated the ideas and provided input on the type and nature of animations, the pain management clinicians primarily led the script writing process along with the animator and health literacy expert due to time constraints. On reflection, allocating more time and funding to the PAG members would have increased the extent of co-design by involving them as ‘co-researchers’ during the content writing process of the animation. The large number of resources created in a relatively short period limited the extent of iterative during the design, such as feedback and consensus; aspects synonymous with a co-design concept.

*Diversity of the PAG and the Māori community hui group may limit acceptability of the content.* There were more female than male participants in both the PAG and the Māori community hui. The pain team did attempt to recruit more male participants, but this was unsuccessful. While persistent pain in Aotearoa is more prevalent in females than males [33], and more females seek support for persistent pain than males, this discrepancy is a limitation of the acceptability of the co-designed contents. The low number of Māori patients referred to tertiary pain services in Aotearoa [5] meant there were a limited number of Māori people with experience of the hospital-based group PMP whom we could recruit into the PAG. The acknowledged discrepancy between high persistent pain prevalence and low health service uptake by Māori patients was one of the key impetus for this project. The inability to recruit Māori patients who had been through the existing pain programme necessitated involvement of Māori community members. While all our Māori participants did live with persistent pain, the implications of this limitation is discussed in more detail below.

*Māori engagement was collaborative with caveats*. The Māori community members acknowledged that this was their first experience of sharing their experiences of persistent pain and valued the iterative engagement [15] and the opportunity to provide input to the online programme to enhance cultural appropriateness and accessibility. Despite these positive comments, we acknowledge that none of the Māori community members had ever been referred to the pain service and thus they were unfamiliar with the service and PMP. This lack of familiarity with what such programmes comprise meant our engagement was limited to how the programme was to be delivered and how to best ensure a Māori participant would feel comfortable when attending this online programme. As our previous evaluations on global pain management websites [27] and apps [26] identified a lack of culturally tailored online information (i.e. websites and apps) for people with persistent pain and no national website for pain management, we used an existing culturally tailored national public website (https://depression.org.nz/maori/) as an example to assist the Māori community members’ understanding of what such websites might look like. The Māori health literacy expert on the project had a background of working with Māori whānau and this ensured the content we generated was easy to read and understand. We also followed recommendations that information provision via short videos and metaphors assists in tailoring information for culturally and linguistically diverse communities living with persistent pain [40].

*Extent of Beta testing was constrained*. All participants, PAG members and Māori community hui participants, had an opportunity to use the online programme and provide feedback. However, as we were time pressed, we could only give them about 3 to 4 weeks to go through all 12 modules. Although participants had a chance to look at most of the resources, there was a large amount of resources (n = 140) for them to go through in a limited time. Future studies should consider providing more time to focus on this aspect. Ideas such as, interactive quizzes, a content progress bar, and specific changes to the design of the online community discussion page were not possible. These were all elements which were ranked lower in preference by the PAG members (Fig. 2). These content delivery ideas were not attainable due to the online platform’s technical flexibility and funding restrictions. PAG members were understanding about these limitations.

## Conclusions

This is the first on-line group pain management programme for people living with persistent pain developed in Aotearoa, a programme with an emphasis on cultural appropriateness for Māori. Working with a small group of PAG members with existing relationships with the researchers and the clinicians enhanced trust and this facilitated the co-design process. Future studies could prioritise more involvement and financial recompense from the PAG and Māori community members to maximise the extent of co-design and co-creation, especially in the beta testing phase. Our study also informs culturally appropriate engagement processes for co-designing pain management interventions for Indigenous and ethnoculturally diverse people living with persistent pain.

## Supplementary Information

**Additional file 1**. The Guidance for Reporting Involvement of Patients and the Public—GRIPP2 checklist for iSelf-help.

**Additional file 2**. Interview guide for patient video stories.

**Additional file 3**. Interview guide for video stories of Māori participants.

## Data Availability

The raw data from the interviews and Nominal Group Technique are available from the corresponding author on request.

## Abbreviations

PAR: Participatory action research
PAG: Patient advisory group
PMP: Pain management programme
