# Supplementary material for: iSelf-Help: a co-designed, culturally appropriate, online pain management programme in Aotearoa
Source: Res Involv Engagem. 2022 Feb 21;8:6. doi: 10.1186/s40900-022-00339-9 (PMC8862515; doi:10.1186/s40900-022-00339-9)
Supplement: Supplementary file 2 — Additional file 2. Interview guide for patient video stories. [file 40900_2022_339_MOESM2_ESM.docx]

# Additional file 2. Interview guide for patient video stories

| **Knowing the person (1-2mins)** |
| --- |
| Tell us a bit about yourself  Tell about your family, culture, values  What sort of person you are?  What do you like to do (home/work/other interests)? |
| **Understanding their pain journey (2-5 mins)** |
| Tell us about your pain journey  How long have you had the pain?  Have you had a specific diagnosis for your pain?  How long is it since your diagnosis? |
| **Impact of completing a pain management programme in CCDHB (10-15mins)** |
| Describe the impact of pain management programme in your life  What did you learn about yourself, your close people and support person  How did living with pain change as a result of the programme?  Feelings of self, others and health professionals  What worked and did not work?  One specific thing which *really made* difference of being in the programme  One specific thing that *did not work* for you from being in the programme |
| **Stories** |
| Can you tell us an experience or share an example of when you had to use these strategies after the programme (e.g.) understanding pain pathways, managing negative thoughts or keeping yourself active (topics from below)?  **Strategies/topics**   - Sensory nervous system and pain pathways - Threat/Stress response - Neurosignatures – role of memory in pain - Exercise - Goalsetting - Sleep - Pacing or scheduling daily activities - Psychological strategies (Feelings of pain, mindfulness, CBT, ACT) - Medication - Supports: Whānau/family and friends* - Communication with health providers   **Prompts**  How it worked?  Why it was challenging?  What motivated to keep going? |
| **Notes**: We plan to interview about 8-10 participants at the CCDHB Pain management service, so to ensure a balance, we’d like them to choose 3-4 topics for the focus of their interview. |
