# Supplementary material for: iSelf-Help: a co-designed, culturally appropriate, online pain management programme in Aotearoa
Source: Res Involv Engagem. 2022 Feb 21;8:6. doi: 10.1186/s40900-022-00339-9 (PMC8862515; doi:10.1186/s40900-022-00339-9)
Supplement: Supplementary file 3 — Additional file 3. Interview guide for video stories of Māori participants. [file 40900_2022_339_MOESM3_ESM.docx]

# Additional file 3. Interview guide for video stories of Māori participants

| **Knowing the person (1-2mins)** |
| --- |
| Tell us a bit about yourself  Tell about your whānau, culture, values  What sort of person you are?  What do you like to do (home/work/other interests)? |
| **Understanding your pain journey (2-5 mins)** |
| Tell us about your pain journey  How long have you had the pain?  Have you had a specific diagnosis for your pain?  How long is it since your diagnosis? |
| **Impact of pain on your ‘self’ and your whānau (10-15mins)** |
| Describe the impact of pain in your life and your whānau  How is it like living with pain?  Feelings of self, others and health professionals |
| **Things that helped to cope with your pain** |
| What worked and did not work?  Any specific thing which *really made* difference to your pain  Any specific thing that *did not work* for you  Do you talk about it to your whānau and friends  **Prompts**  Activities (Waiata, going to Marae)  Prayer  Medication  Communication with health providers |
| **For support people** |
| Tell us your experience of supporting your whānau with pain?  a. How difficult it is to talk about pain to your whānau with pain? |
| **General prompts**  How it worked?  Why it was challenging?  What motivated to keep going? |
